# Supplementary material for: Conservation of Sex-Linked Markers among Conspecific Populations of a Viviparous Skink, Niveoscincus ocellatus, Exhibiting Genetic and Temperature-Dependent Sex Determination
Source: Genome Biol Evol. 2018 Mar 5;10(4):1079–87. doi: 10.1093/gbe/evy042 (PMC5905450; doi:10.1093/gbe/evy042)
Supplement: Supplementary Materials [file evy042_supp.docx]

**Table S1. Sex linked Presence / Absence loci in *Niveoscincus ocellatus*.** Proportion of presence / absence genotypes in males and females common to both highland and lowland populations and unique to each population of *Niveoscincus* *ocellatus*. Score of 1 denotes presence of the fragment, 0 denotes absence of the fragment and ‘-‘ denotes a putative heterozygous score for the fragment. All loci denote XY heterogamety with the exception of locus marked **^*^**.

| Common loci |  | Males | | |  | Females | | |  | Fishers p value |
| --- | --- | --- | --- | --- | --- | --- | --- | --- | --- | --- |
|  |  | Prop. ‘1’ | Prop. '-' | Prop. '0' |  | Prop. '1' | Prop. '-' | Prop. ‘0’ |  |  |
| NO27479 |  | 1.00 | 0.00 | 0.00 |  | 0.07 | 0.00 | 0.93 |  | <0.001 |
| NO25981 |  | 0.98 | 0.02 | 0.00 |  | 0.00 | 0.00 | 1.00 |  | <0.001 |
| NO17412 |  | 0.96 | 0.04 | 0.00 |  | 0.00 | 0.02 | 0.98 |  | <0.001 |
| NO13113 |  | 0.94 | 0.06 | 0.00 |  | 0.00 | 0.00 | 1.00 |  | <0.001 |
| NO11735 |  | 0.91 | 0.09 | 0.00 |  | 0.00 | 0.00 | 1.00 |  | <0.001 |
| NO09026 |  | 0.94 | 0.06 | 0.00 |  | 0.00 | 0.00 | 1.00 |  | <0.001 |
| NO06180 |  | 0.96 | 0.04 | 0.00 |  | 0.00 | 0.00 | 1.00 |  | <0.001 |
| NO06174 |  | 0.91 | 0.09 | 0.00 |  | 0.00 | 0.00 | 1.00 |  | <0.001 |
| NO06148 |  | 0.98 | 0.02 | 0.00 |  | 0.00 | 0.00 | 1.00 |  | <0.001 |
| NO04112 |  | 0.94 | 0.06 | 0.00 |  | 0.00 | 0.00 | 1.00 |  | <0.001 |
| NO04111 |  | 0.81 | 0.17 | 0.02 |  | 0.00 | 0.00 | 1.00 |  | <0.001 |
| NO04106 |  | 0.96 | 0.04 | 0.00 |  | 0.00 | 0.00 | 1.00 |  | <0.001 |
| NO01619 |  | 0.91 | 0.09 | 0.00 |  | 0.00 | 0.00 | 1.00 |  | <0.001 |
| NO01528 |  | 0.91 | 0.09 | 0.00 |  | 0.00 | 0.00 | 1.00 |  | <0.001 |
| NO00798 |  | 0.98 | 0.02 | 0.00 |  | 0.00 | 0.00 | 1.00 |  | <0.001 |
| NO00674 |  | 0.87 | 0.13 | 0.00 |  | 0.00 | 0.00 | 1.00 |  | <0.001 |
| NO99977 |  | 0.94 | 0.06 | 0.00 |  | 0.00 | 0.02 | 0.98 |  | <0.001 |
| NO99347 |  | 0.96 | 0.02 | 0.02 |  | 0.00 | 0.00 | 1.00 |  | <0.001 |
| NO98718 |  | 0.94 | 0.06 | 0.00 |  | 0.00 | 0.00 | 1.00 |  | <0.001 |
| NO98519 |  | 0.98 | 0.02 | 0.00 |  | 0.00 | 0.00 | 1.00 |  | <0.001 |
| NO98495 |  | 0.96 | 0.04 | 0.00 |  | 0.00 | 0.00 | 1.00 |  | <0.001 |
| NO95026 |  | 0.98 | 0.02 | 0.00 |  | 0.00 | 0.02 | 0.98 |  | <0.001 |
| NO95024 |  | 0.98 | 0.02 | 0.00 |  | 0.00 | 0.02 | 0.98 |  | <0.001 |

| Common loci |  | Males | | |  | Females | | |  | Fishers p value |
| --- | --- | --- | --- | --- | --- | --- | --- | --- | --- | --- |
|  |  | Prop. ‘1’ | Prop. '-' | Prop. '0' |  | Prop. '1' | Prop. '-' | Prop. ‘0’ |  |  |
| NO95017 |  | 0.89 | 0.11 | 0.00 |  | 0.00 | 0.00 | 1.00 |  | <0.001 |
| NO94951 |  | 0.98 | 0.02 | 0.00 |  | 0.00 | 0.00 | 1.00 |  | <0.001 |
| NO94946 |  | 0.96 | 0.04 | 0.00 |  | 0.00 | 0.00 | 1.00 |  | <0.001 |
| NO94938 |  | 0.96 | 0.04 | 0.00 |  | 0.00 | 0.00 | 1.00 |  | <0.001 |
| NO94918 |  | 1.00 | 0.00 | 0.00 |  | 0.00 | 0.00 | 1.00 |  | <0.001 |
| NO94907 |  | 0.98 | 0.02 | 0.00 |  | 0.00 | 0.02 | 0.98 |  | <0.001 |
| NO94905 |  | 0.94 | 0.06 | 0.00 |  | 0.00 | 0.00 | 1.00 |  | <0.001 |
| NO94890 |  | 0.98 | 0.02 | 0.00 |  | 0.00 | 0.00 | 1.00 |  | <0.001 |
| NO94882 |  | 0.83 | 0.04 | 0.13 |  | 0.00 | 0.00 | 1.00 |  | <0.001 |
| NO94872 |  | 0.98 | 0.02 | 0.00 |  | 0.00 | 0.00 | 1.00 |  | <0.001 |
| NO94858 |  | 0.94 | 0.04 | 0.02 |  | 0.00 | 0.00 | 1.00 |  | <0.001 |
| NO94833 |  | 1.00 | 0.00 | 0.00 |  | 0.00 | 0.00 | 1.00 |  | <0.001 |
| NO94820 |  | 0.91 | 0.09 | 0.00 |  | 0.00 | 0.00 | 1.00 |  | <0.001 |
| NO94818 |  | 0.96 | 0.04 | 0.00 |  | 0.00 | 0.00 | 1.00 |  | <0.001 |
| NO94817 |  | 0.89 | 0.09 | 0.02 |  | 0.00 | 0.00 | 1.00 |  | <0.001 |
| NO94803 |  | 0.83 | 0.17 | 0.00 |  | 0.00 | 0.00 | 1.00 |  | <0.001 |
| NO94785 |  | 0.96 | 0.04 | 0.00 |  | 0.00 | 0.00 | 1.00 |  | <0.001 |
| NO94771 |  | 0.94 | 0.06 | 0.00 |  | 0.00 | 0.00 | 1.00 |  | <0.001 |
| NO94762 |  | 0.87 | 0.13 | 0.00 |  | 0.00 | 0.00 | 1.00 |  | <0.001 |
| NO94757 |  | 0.89 | 0.11 | 0.00 |  | 0.00 | 0.00 | 1.00 |  | <0.001 |
| NO94726 |  | 0.83 | 0.17 | 0.00 |  | 0.00 | 0.00 | 1.00 |  | <0.001 |
| NO94717 |  | 1.00 | 0.00 | 0.00 |  | 0.00 | 0.00 | 1.00 |  | <0.001 |
| NO94701 |  | 0.91 | 0.09 | 0.00 |  | 0.00 | 0.00 | 1.00 |  | <0.001 |
| NO94680 |  | 0.94 | 0.06 | 0.00 |  | 0.00 | 0.00 | 1.00 |  | <0.001 |
| NO94670 |  | 0.91 | 0.09 | 0.00 |  | 0.00 | 0.00 | 1.00 |  | <0.001 |
| NO94660 |  | 0.89 | 0.11 | 0.00 |  | 0.00 | 0.00 | 1.00 |  | <0.001 |
| NO94656 |  | 0.94 | 0.06 | 0.00 |  | 0.00 | 0.00 | 1.00 |  | <0.001 |

| Common loci |  | Males | | |  | Females | | |  | Fishers p value |
| --- | --- | --- | --- | --- | --- | --- | --- | --- | --- | --- |
|  |  | Prop. ‘1’ | Prop. '-' | Prop. '0' |  | Prop. '1' | Prop. '-' | Prop. ‘0’ |  |  |
| NO94646 |  | 0.94 | 0.06 | 0.00 |  | 0.00 | 0.00 | 1.00 |  | <0.001 |
| NO94601 |  | 0.96 | 0.04 | 0.00 |  | 0.00 | 0.00 | 1.00 |  | <0.001 |
| NO94543 |  | 0.91 | 0.09 | 0.00 |  | 0.00 | 0.00 | 1.00 |  | <0.001 |
| NO94449 |  | 0.98 | 0.02 | 0.00 |  | 0.00 | 0.00 | 1.00 |  | <0.001 |
| NO94291 |  | 0.96 | 0.04 | 0.00 |  | 0.00 | 0.00 | 1.00 |  | <0.001 |
| NO95025 |  | 0.94 | 0.06 | 0.00 |  | 0.00 | 0.02 | 0.98 |  | <0.001 |
| NO94980 |  | 1.00 | 0.00 | 0.00 |  | 0.00 | 0.05 | 0.95 |  | <0.001 |
| NO94948 |  | 0.96 | 0.04 | 0.00 |  | 0.00 | 0.02 | 0.98 |  | <0.001 |
| NO94939 |  | 0.96 | 0.04 | 0.00 |  | 0.00 | 0.02 | 0.98 |  | <0.001 |
| NO27293 |  | 0.94 | 0.06 | 0.00 |  | 0.00 | 0.00 | 1.00 |  | <0.001 |
| NO27141 |  | 0.91 | 0.09 | 0.00 |  | 0.00 | 0.00 | 1.00 |  | <0.001 |
| NO25915 |  | 0.87 | 0.13 | 0.00 |  | 0.00 | 0.00 | 1.00 |  | <0.001 |
| NO22758 |  | 0.87 | 0.13 | 0.00 |  | 0.00 | 0.00 | 1.00 |  | <0.001 |
| NO20991 |  | 0.87 | 0.13 | 0.00 |  | 0.00 | 0.00 | 1.00 |  | <0.001 |
| NO18137 |  | 0.96 | 0.04 | 0.00 |  | 0.00 | 0.00 | 1.00 |  | <0.001 |
| NO15686 |  | 0.87 | 0.13 | 0.00 |  | 0.00 | 0.00 | 1.00 |  | <0.001 |
| NO08370 |  | 0.89 | 0.11 | 0.00 |  | 0.00 | 0.00 | 1.00 |  | <0.001 |
| NO07276 |  | 0.94 | 0.06 | 0.00 |  | 0.00 | 0.00 | 1.00 |  | <0.001 |
| NO06426 |  | 0.91 | 0.09 | 0.00 |  | 0.00 | 0.00 | 1.00 |  | <0.001 |
| NO04817 |  | 0.85 | 0.15 | 0.00 |  | 0.00 | 0.02 | 0.98 |  | <0.001 |
| NO04179 |  | 0.85 | 0.15 | 0.00 |  | 0.00 | 0.00 | 1.00 |  | <0.001 |
| NO04137 |  | 0.96 | 0.04 | 0.00 |  | 0.00 | 0.00 | 1.00 |  | <0.001 |
| NO04128 |  | 0.85 | 0.15 | 0.00 |  | 0.00 | 0.00 | 1.00 |  | <0.001 |
| NO04085 |  | 0.94 | 0.06 | 0.00 |  | 0.00 | 0.02 | 0.98 |  | <0.001 |
| NO04068 |  | 0.85 | 0.15 | 0.00 |  | 0.00 | 0.00 | 1.00 |  | <0.001 |
| NO01662 |  | 0.89 | 0.11 | 0.00 |  | 0.00 | 0.00 | 1.00 |  | <0.001 |
| NO00349 |  | 0.89 | 0.11 | 0.00 |  | 0.00 | 0.00 | 1.00 |  | <0.001 |

| Common loci |  | Males | | |  | Females | | |  | Fishers p value |
| --- | --- | --- | --- | --- | --- | --- | --- | --- | --- | --- |
|  |  | Prop. ‘1’ | Prop. '-' | Prop. '0' |  | Prop. '1' | Prop. '-' | Prop. ‘0’ |  |  |
| NO99580 |  | 0.89 | 0.11 | 0.00 |  | 0.00 | 0.00 | 1.00 |  | <0.001 |
| NO95016 |  | 0.94 | 0.06 | 0.00 |  | 0.00 | 0.00 | 1.00 |  | <0.001 |
| NO94991 |  | 0.94 | 0.06 | 0.00 |  | 0.00 | 0.00 | 1.00 |  | <0.001 |
| NO94975 |  | 0.91 | 0.09 | 0.00 |  | 0.00 | 0.00 | 1.00 |  | <0.001 |
| NO94972 |  | 0.91 | 0.09 | 0.00 |  | 0.00 | 0.00 | 1.00 |  | <0.001 |
| NO94965 |  | 0.91 | 0.09 | 0.00 |  | 0.00 | 0.00 | 1.00 |  | <0.001 |
| NO94947 |  | 0.94 | 0.06 | 0.00 |  | 0.00 | 0.02 | 0.98 |  | <0.001 |
| NO94943 |  | 0.96 | 0.04 | 0.00 |  | 0.00 | 0.02 | 0.98 |  | <0.001 |
| NO94934 |  | 0.87 | 0.13 | 0.00 |  | 0.00 | 0.00 | 1.00 |  | <0.001 |
| NO94928 |  | 0.91 | 0.09 | 0.00 |  | 0.00 | 0.00 | 1.00 |  | <0.001 |
| NO94921 |  | 0.87 | 0.13 | 0.00 |  | 0.00 | 0.00 | 1.00 |  | <0.001 |
| NO94902 |  | 0.91 | 0.09 | 0.00 |  | 0.00 | 0.00 | 1.00 |  | <0.001 |
| NO94880 |  | 0.94 | 0.06 | 0.00 |  | 0.00 | 0.00 | 1.00 |  | <0.001 |
| NO94865 |  | 0.87 | 0.13 | 0.00 |  | 0.00 | 0.00 | 1.00 |  | <0.001 |
| NO94860 |  | 0.81 | 0.19 | 0.00 |  | 0.00 | 0.00 | 1.00 |  | <0.001 |
| NO94847 |  | 0.91 | 0.09 | 0.00 |  | 0.00 | 0.00 | 1.00 |  | <0.001 |
| NO94828 |  | 0.89 | 0.11 | 0.00 |  | 0.00 | 0.00 | 1.00 |  | <0.001 |
| NO94825 |  | 0.83 | 0.17 | 0.00 |  | 0.00 | 0.00 | 1.00 |  | <0.001 |
| NO94824 |  | 0.91 | 0.09 | 0.00 |  | 0.00 | 0.00 | 1.00 |  | <0.001 |
| NO94815 |  | 0.91 | 0.09 | 0.00 |  | 0.00 | 0.00 | 1.00 |  | <0.001 |
| NO94806 |  | 0.85 | 0.15 | 0.00 |  | 0.00 | 0.00 | 1.00 |  | <0.001 |
| NO94799 |  | 0.94 | 0.06 | 0.00 |  | 0.00 | 0.00 | 1.00 |  | <0.001 |
| NO94791 |  | 0.83 | 0.17 | 0.00 |  | 0.00 | 0.00 | 1.00 |  | <0.001 |
| NO94759 |  | 0.94 | 0.06 | 0.00 |  | 0.00 | 0.02 | 0.98 |  | <0.001 |
| NO94758 |  | 0.89 | 0.11 | 0.00 |  | 0.00 | 0.02 | 0.98 |  | <0.001 |
| NO94756 |  | 0.94 | 0.06 | 0.00 |  | 0.00 | 0.00 | 1.00 |  | <0.001 |
| NO94739 |  | 0.85 | 0.15 | 0.00 |  | 0.00 | 0.00 | 1.00 |  | <0.001 |

| Common loci |  | Males | | |  | Females | | |  | Fishers p value |
| --- | --- | --- | --- | --- | --- | --- | --- | --- | --- | --- |
|  |  | Prop. ‘1’ | Prop. '-' | Prop. '0' |  | Prop. '1' | Prop. '-' | Prop. ‘0’ |  |  |
| NO94721 |  | 0.91 | 0.09 | 0.00 |  | 0.00 | 0.00 | 1.00 |  | <0.001 |
| NO94702 |  | 0.85 | 0.13 | 0.02 |  | 0.00 | 0.00 | 1.00 |  | <0.001 |
| NO94699 |  | 0.89 | 0.11 | 0.00 |  | 0.00 | 0.00 | 1.00 |  | <0.001 |
| NO94694 |  | 0.94 | 0.06 | 0.00 |  | 0.00 | 0.00 | 1.00 |  | <0.001 |
| NO94675 |  | 0.87 | 0.13 | 0.00 |  | 0.00 | 0.00 | 1.00 |  | <0.001 |
| NO94667 |  | 0.91 | 0.09 | 0.00 |  | 0.00 | 0.00 | 1.00 |  | <0.001 |
| NO94640 |  | 0.94 | 0.06 | 0.00 |  | 0.00 | 0.00 | 1.00 |  | <0.001 |
| NO94627 |  | 0.91 | 0.09 | 0.00 |  | 0.00 | 0.00 | 1.00 |  | <0.001 |
| NO94605 |  | 0.85 | 0.15 | 0.00 |  | 0.00 | 0.00 | 1.00 |  | <0.001 |
| NO94585 |  | 0.89 | 0.11 | 0.00 |  | 0.00 | 0.00 | 1.00 |  | <0.001 |
| NO94564 |  | 0.89 | 0.11 | 0.00 |  | 0.00 | 0.00 | 1.00 |  | <0.001 |
| NO94528 |  | 0.94 | 0.06 | 0.00 |  | 0.00 | 0.00 | 1.00 |  | <0.001 |
| NO94526 |  | 0.89 | 0.11 | 0.00 |  | 0.00 | 0.00 | 1.00 |  | <0.001 |
| NO26343 |  | 0.91 | 0.09 | 0.00 |  | 0.00 | 0.02 | 0.98 |  | <0.001 |
| NO23032 |  | 0.89 | 0.11 | 0.00 |  | 0.00 | 0.00 | 1.00 |  | <0.001 |
| NO10616 |  | 0.87 | 0.13 | 0.00 |  | 0.00 | 0.00 | 1.00 |  | <0.001 |
| NO08457 |  | 0.85 | 0.15 | 0.00 |  | 0.00 | 0.00 | 1.00 |  | <0.001 |
| NO04193 |  | 0.85 | 0.15 | 0.00 |  | 0.00 | 0.00 | 1.00 |  | <0.001 |
| NO94976 |  | 0.89 | 0.11 | 0.00 |  | 0.00 | 0.00 | 1.00 |  | <0.001 |
| NO94810 |  | 0.87 | 0.13 | 0.00 |  | 0.00 | 0.00 | 1.00 |  | <0.001 |
| NO94794 |  | 0.85 | 0.15 | 0.00 |  | 0.00 | 0.00 | 1.00 |  | <0.001 |
| NO94779 |  | 0.91 | 0.09 | 0.00 |  | 0.00 | 0.00 | 1.00 |  | <0.001 |
| NO94695 |  | 0.87 | 0.13 | 0.00 |  | 0.00 | 0.02 | 0.98 |  | <0.001 |
| NO94665 |  | 0.89 | 0.11 | 0.00 |  | 0.00 | 0.00 | 1.00 |  | <0.001 |
| NO94599 |  | 0.87 | 0.13 | 0.00 |  | 0.00 | 0.00 | 1.00 |  | <0.001 |
| NO94566 |  | 0.87 | 0.13 | 0.00 |  | 0.00 | 0.00 | 1.00 |  | <0.001 |
| NO20597 |  | 0.85 | 0.15 | 0.00 |  | 0.00 | 0.02 | 0.98 |  | <0.001 |

| Common loci |  | Males | | |  | Females | | |  | Fishers p value |
| --- | --- | --- | --- | --- | --- | --- | --- | --- | --- | --- |
|  |  | Prop. ‘1’ | Prop. '-' | Prop. '0' |  | Prop. '1' | Prop. '-' | Prop. ‘0’ |  |  |
| NO01905 |  | 0.85 | 0.15 | 0.00 |  | 0.00 | 0.00 | 1.00 |  | <0.001 |
| NO95027 |  | 0.83 | 0.17 | 0.00 |  | 0.00 | 0.00 | 1.00 |  | <0.001 |
| NO94987 |  | 0.83 | 0.17 | 0.00 |  | 0.00 | 0.00 | 1.00 |  | <0.001 |
| NO94689 |  | 0.81 | 0.19 | 0.00 |  | 0.00 | 0.00 | 1.00 |  | <0.001 |
| NO94527 |  | 0.83 | 0.17 | 0.00 |  | 0.00 | 0.00 | 1.00 |  | <0.001 |
| NO94518 |  | 0.89 | 0.11 | 0.00 |  | 0.00 | 0.00 | 1.00 |  | <0.001 |
| NO94766 |  | 0.89 | 0.06 | 0.04 |  | 0.00 | 0.00 | 1.00 |  | <0.001 |
| NO94749 |  | 0.89 | 0.09 | 0.02 |  | 0.00 | 0.00 | 1.00 |  | <0.001 |
| NO27615 |  | 0.98 | 0.02 | 0.00 |  | 0.12 | 0.02 | 0.85 |  | <0.001 |
| NO94903 |  | 0.81 | 0.19 | 0.00 |  | 0.00 | 0.00 | 1.00 |  | <0.001 |
| NO94881 |  | 0.87 | 0.13 | 0.00 |  | 0.00 | 0.00 | 1.00 |  | <0.001 |
| NO92667 |  | 0.98 | 0.02 | 0.00 |  | 0.02 | 0.15 | 0.83 |  | <0.001 |
| NO94732 |  | 0.87 | 0.11 | 0.02 |  | 0.00 | 0.00 | 1.00 |  | <0.001 |
| NO94530 |  | 0.87 | 0.11 | 0.02 |  | 0.00 | 0.00 | 1.00 |  | <0.001 |
| NO94666 |  | 0.83 | 0.15 | 0.02 |  | 0.00 | 0.00 | 1.00 |  | <0.001 |
| NO94941 |  | 0.79 | 0.19 | 0.02 |  | 0.00 | 0.00 | 1.00 |  | <0.001 |
| NO94539 |  | 0.79 | 0.15 | 0.06 |  | 0.00 | 0.00 | 1.00 |  | <0.001 |
| NO01037 |  | 0.98 | 0.02 | 0.00 |  | 0.05 | 0.20 | 0.76 |  | <0.001 |
| NO17728 |  | 1.00 | 0.00 | 0.00 |  | 0.10 | 0.02 | 0.88 |  | <0.001 |
| NO94843 |  | 0.91 | 0.00 | 0.09 |  | 0.20 | 0.00 | 0.80 |  | <0.001 |
| NO18471 |  | 0.94 | 0.04 | 0.02 |  | 0.22 | 0.05 | 0.73 |  | 0.002 |
| Highland loci |  | Males | | |  | Females | | |  | Fishers p value |
|  |  | Prop. ‘1’ | Prop. '-' | Prop. '0' |  | Prop. '1' | Prop. '-' | Prop. ‘0’ |  |  |
| NO26788 |  | 0.96 | 0.04 | 0.00 |  | 0.00 | 0.00 | 1.00 |  | <0.001 |
| NO12766 |  | 1.00 | 0.00 | 0.00 |  | 0.00 | 0.00 | 1.00 |  | <0.001 |
| NO95824 |  | 1.00 | 0.00 | 0.00 |  | 0.00 | 0.00 | 1.00 |  | <0.001 |
| NO95537 |  | 0.96 | 0.04 | 0.00 |  | 0.00 | 0.00 | 1.00 |  | <0.001 |

| Highland loci |  | Males | | |  | Females | | |  | Fishers p value |
| --- | --- | --- | --- | --- | --- | --- | --- | --- | --- | --- |
|  |  | Prop. ‘1’ | Prop. '-' | Prop. '0' |  | Prop. '1' | Prop. '-' | Prop. ‘0’ |  |  |
| NO95785 |  | 1.00 | 0.00 | 0.00 |  | 0.00 | 0.05 | 0.95 |  | <0.001 |
| NO04320 |  | 0.87 | 0.13 | 0.00 |  | 0.00 | 0.00 | 1.00 |  | <0.001 |
| NO95729 |  | 0.87 | 0.13 | 0.00 |  | 0.00 | 0.00 | 1.00 |  | <0.001 |
| NO95674 |  | 0.91 | 0.09 | 0.00 |  | 0.00 | 0.00 | 1.00 |  | <0.001 |
| NO95621 |  | 0.91 | 0.09 | 0.00 |  | 0.00 | 0.00 | 1.00 |  | <0.001 |
| NO92926 |  | 0.96 | 0.00 | 0.04 |  | 0.00 | 0.00 | 1.00 |  | <0.001 |
| NO25902 |  | 1.00 | 0.00 | 0.00 |  | 0.05 | 0.05 | 0.90 |  | <0.001 |
| NO13170 |  | 0.35 | 0.65 | 0.00 |  | 0.00 | 0.00 | 1.00 |  | <0.001 |
| NO09552 |  | 0.61 | 0.00 | 0.39 |  | 0.00 | 0.00 | 1.00 |  | 0.001 |
| NO00517 |  | 0.74 | 0.26 | 0.00 |  | 0.19 | 0.29 | 0.52 |  | 0.001 |
| NO04304 |  | 1.00 | 0.00 | 0.00 |  | 0.00 | 0.00 | 1.00 |  | <0.001 |
| NO25722 |  | 1.00 | 0.00 | 0.00 |  | 0.38 | 0.10 | 0.52 |  | 0.001 |
| Lowland loci |  | Males | | |  | Females | | |  | Fishers p value |
|  |  | Prop. ‘1’ | Prop. '-' | Prop. '0' |  | Prop. '1' | Prop. '-' | Prop. ‘0’ |  |  |
| NO94625 |  | 0.96 | 0.04 | 0.00 |  | 0.00 | 0.00 | 1.00 |  | <0.001 |
| NO95018 |  | 1.00 | 0.00 | 0.00 |  | 0.00 | 0.05 | 0.95 |  | <0.001 |
| NO94661 |  | 0.96 | 0.04 | 0.00 |  | 0.00 | 0.05 | 0.95 |  | <0.001 |
| NO24878 |  | 0.96 | 0.00 | 0.04 |  | 0.00 | 0.00 | 1.00 |  | <0.001 |
| NO26368 |  | 0.83 | 0.17 | 0.00 |  | 0.00 | 0.00 | 1.00 |  | <0.001 |
| NO94893 |  | 0.83 | 0.17 | 0.00 |  | 0.00 | 0.00 | 1.00 |  | <0.001 |
| NO27480 |  | 0.92 | 0.04 | 0.04 |  | 0.00 | 0.00 | 1.00 |  | <0.001 |
| NO12765 |  | 0.92 | 0.04 | 0.04 |  | 0.00 | 0.00 | 1.00 |  | <0.001 |
| NO27095 |  | 0.88 | 0.04 | 0.08 |  | 0.00 | 0.00 | 1.00 |  | <0.001 |
| NO23045 |  | 0.88 | 0.04 | 0.08 |  | 0.00 | 0.00 | 1.00 |  | <0.001 |
| NO94900 |  | 0.92 | 0.00 | 0.08 |  | 0.00 | 0.00 | 1.00 |  | <0.001 |
| NO94542 |  | 0.92 | 0.00 | 0.08 |  | 0.00 | 0.00 | 1.00 |  | <0.001 |
| NO94648 |  | 0.83 | 0.08 | 0.08 |  | 0.00 | 0.00 | 1.00 |  | <0.001 |

| Lowland loci |  | Males | | |  | Females | | |  | Fishers p value |
| --- | --- | --- | --- | --- | --- | --- | --- | --- | --- | --- |
|  |  | Prop. ‘1’ | Prop. '-' | Prop. '0' |  | Prop. '1' | Prop. '-' | Prop. ‘0’ |  |  |
| NO24877 |  | 0.83 | 0.00 | 0.17 |  | 0.00 | 0.00 | 1.00 |  | <0.001 |
| NO01508 |  | 0.58 | 0.38 | 0.04 |  | 0.00 | 0.00 | 1.00 |  | <0.001 |
| NO17727 |  | 1.00 | 0.00 | 0.00 |  | 0.20 | 0.05 | 0.75 |  | <0.001 |
| NO00397 |  | 0.79 | 0.21 | 0.00 |  | 0.20 | 0.15 | 0.65 |  | <0.001 |
| NO95000 |  | 0.75 | 0.00 | 0.25 |  | 0.05 | 0.05 | 0.90 |  | <0.001 |
| NO90328 |  | 0.63 | 0.38 | 0.00 |  | 0.20 | 0.15 | 0.65 |  | 0.001 |
| NO93463**^*^** |  | 0.00 | 0.00 | 1.00 |  | 0.55 | 0.00 | 0.45 |  | 0.002 |

**Table S2. Proportion of genotypes in males and females for SNPs with alleles that segregate with sex according to the ‘null exclusive’ sex-linked model in populations of *Niveoscincus* *ocellatus*.** Score of ‘2’ indicates heterozygous for the reference and SNP allele, ‘1’ homozygous for the SNP allele, ‘0’ homozygous for the reference allele,’-‘ homozygous for a null genotype. Alleles of all loci assort according to XY heterogamety. Fig 2 refers to labelling of these loci in figure 2 of manuscript.

| Common  Loci | Fig 2 | REF > SNP | Males | | | | Females | | | | p value |
| --- | --- | --- | --- | --- | --- | --- | --- | --- | --- | --- | --- |
|  |  |  | Prop. ‘2’ | Prop. '1' | Prop. '0' | Prop. '-' | Prop. '2' | Prop. '1' | Prop. ‘0’ | Prop. ‘-’ |  |
| NO02579 | a | G>T | 0.98 | 0.02 | 0.00 | 0.00 | 0.00 | 0.00 | 1.00 | 0.00 | <0.001 |
| NO17128 | b | G>A | 0.94 | 0.06 | 0.00 | 0.00 | 0.00 | 0.00 | 1.00 | 0.00 | <0.001 |
| NO02507 | c | C>A | 0.96 | 0.04 | 0.00 | 0.00 | 0.00 | 0.00 | 1.00 | 0.00 | <0.001 |
| NO02972 | d | T>G | 0.94 | 0.06 | 0.00 | 0.00 | 0.00 | 0.00 | 1.00 | 0.00 | <0.001 |
| NO27498 | e | C>T | 0.96 | 0.04 | 0.00 | 0.00 | 0.02 | 0.00 | 0.98 | 0.00 | <0.001 |
| NO20596 | f | T>C | 0.98 | 0.02 | 0.00 | 0.00 | 0.00 | 0.00 | 1.00 | 0.00 | <0.001 |
| NO18136 | g | C>T | 0.98 | 0.02 | 0.00 | 0.00 | 0.00 | 0.00 | 1.00 | 0.00 | <0.001 |
| NO00135 | h | C>T | 0.98 | 0.02 | 0.00 | 0.00 | 0.00 | 0.00 | 1.00 | 0.00 | <0.001 |
| NO05744 | i | C>T | 0.62 | 0.36 | 0.00 | 0.02 | 0.00 | 0.00 | 0.93 | 0.07 | <0.001 |
| NO08456 | j | A>G | 0.89 | 0.11 | 0.00 | 0.00 | 0.00 | 0.00 | 0.98 | 0.02 | <0.001 |
| NO10615 | k | G>A | 0.89 | 0.11 | 0.00 | 0.00 | 0.00 | 0.00 | 0.98 | 0.02 | <0.001 |
| NO18978 | l | A>G | 0.83 | 0.15 | 0.02 | 0.00 | 0.02 | 0.00 | 0.98 | 0.00 | <0.001 |
| NO25979 | m | G>A | 0.83 | 0.17 | 0.00 | 0.00 | 0.00 | 0.00 | 0.98 | 0.02 | <0.001 |
| NO07275 | n | C>A | 0.94 | 0.06 | 0.00 | 0.00 | 0.00 | 0.00 | 0.98 | 0.02 | <0.001 |
| NO25980 | o | C>T | 0.57 | 0.43 | 0.00 | 0.00 | 0.00 | 0.00 | 0.76 | 0.24 | <0.001 |
| NO98433 | p | G>A | 0.94 | 0.02 | 0.04 | 0.00 | 0.05 | 0.00 | 0.95 | 0.00 | <0.001 |
| NO06949 | q | T>C | 0.91 | 0.04 | 0.04 | 0.00 | 0.05 | 0.00 | 0.95 | 0.00 | <0.001 |
| NO27225 | r | G>C | 0.77 | 0.21 | 0.02 | 0.00 | 0.00 | 0.00 | 0.90 | 0.10 | <0.001 |
| NO09041 | s | G>A | 0.72 | 0.28 | 0.00 | 0.00 | 0.00 | 0.00 | 0.88 | 0.12 | <0.001 |
| NO01515 | t | A>G | 0.47 | 0.51 | 0.00 | 0.02 | 0.00 | 0.02 | 0.56 | 0.41 | <0.001 |
| NO98768 | u | T>A | 0.72 | 0.09 | 0.17 | 0.02 | 0.00 | 0.00 | 0.93 | 0.07 | <0.001 |
| Highland  Loci | Fig 2 | REF > SNP | Males | | | | Females | | | | p value |
|  |  |  | Prop. ‘2’ | Prop. '1' | Prop. '0' | Prop. '-' | Prop. '2' | Prop. '1' | Prop. ‘0’ | Prop. ‘-’ |  |
| NO12147 | na | C>T | 1.00 | 0.00 | 0.00 | 0.00 | 0.00 | 1.00 | 0.00 | 0.00 | <0.001 |
| NO12353 | na | A>G | 1.00 | 0.00 | 0.00 | 0.00 | 0.00 | 0.00 | 1.00 | 0.00 | <0.001 |
| NO10335 | na | G>A | 0.91 | 0.09 | 0.00 | 0.00 | 0.00 | 0.00 | 1.00 | 0.00 | <0.001 |
| NO27290 | na | G>A | 0.57 | 0.43 | 0.00 | 0.00 | 0.00 | 0.00 | 0.95 | 0.05 | <0.001 |
| NO08127 | na | A>G | 0.70 | 0.00 | 0.30 | 0.00 | 0.43 | 0.57 | 0.00 | 0.00 | 0.002 |
| NO11944 | na | T>C | 0.74 | 0.26 | 0.00 | 0.00 | 0.19 | 0.10 | 0.71 | 0.00 | 0.002 |
| NO02387 | na | C>A | 0.91 | 0.09 | 0.00 | 0.00 | 0.29 | 0.00 | 0.71 | 0.00 | 0.002 |
| NO98731 | na | C>A | 0.78 | 0.00 | 0.22 | 0.00 | 0.29 | 0.67 | 0.05 | 0.00 | 0.001 |
| NO01508 | na | A>G | 1.00 | 0.00 | 0.00 | 0.00 | 0.00 | 0.00 | 1.00 | 0.00 | <0.001 |
| NO01904 | na | A>T | 0.96 | 0.04 | 0.00 | 0.00 | 0.00 | 0.00 | 1.00 | 0.00 | <0.001 |
| NO12765 | na | A>G | 0.17 | 0.83 | 0.00 | 0.00 | 0.00 | 0.00 | 0.62 | 0.38 | <0.001 |
| NO05305 | na | G>A | 0.87 | 0.09 | 0.04 | 0.00 | 0.24 | 0.05 | 0.71 | 0.00 | 0.002 |
| Lowland  Loci | Fig 2 | REF > SNP | Males | | | | Females | | | | p value |
|  |  |  | Prop. ‘2’ | Prop. '1' | Prop. '0' | Prop. '-' | Prop. '2' | Prop. '1' | Prop. ‘0’ | Prop. ‘-’ |  |
| NO98539 | na | G>T | 1.00 | 0.00 | 0.00 | 0.00 | 0.00 | 1.00 | 0.00 | 0.00 | <0.001 |
| NO14859 | na | G>A | 0.46 | 0.33 | 0.17 | 0.04 | 0.00 | 0.00 | 0.80 | 0.20 | <0.001 |
| NO17302 | na | C>T | 1.00 | 0.00 | 0.00 | 0.00 | 0.00 | 1.00 | 0.00 | 0.00 | <0.001 |
| NO05269 | na | G>A | 1.00 | 0.00 | 0.00 | 0.00 | 0.00 | 1.00 | 0.00 | 0.00 | <0.001 |
| NO13601 | na | A>C | 0.58 | 0.42 | 0.00 | 0.00 | 0.00 | 0.00 | 0.90 | 0.10 | <0.001 |
| NO07509 | na | G>A | 0.92 | 0.04 | 0.04 | 0.00 | 0.00 | 0.00 | 1.00 | 0.00 | <0.001 |
| NO16130 | na | C>T | 0.83 | 0.08 | 0.08 | 0.00 | 0.00 | 0.00 | 1.00 | 0.00 | <0.001 |
| NO00420 | na | G>T | 0.88 | 0.08 | 0.04 | 0.00 | 0.00 | 1.00 | 0.00 | 0.00 | <0.001 |
| NO02053 | na | G>A | 0.88 | 0.08 | 0.04 | 0.00 | 0.00 | 1.00 | 0.00 | 0.00 | <0.001 |
| NO98391 | na | C>T | 0.88 | 0.00 | 0.13 | 0.00 | 0.25 | 0.70 | 0.05 | 0.00 | <0.001 |
| NO00397 | na | C>G | 0.79 | 0.00 | 0.21 | 0.00 | 0.30 | 0.65 | 0.05 | 0.00 | 0.002 |
| NO11068 | na | A>G | 0.08 | 0.79 | 0.00 | 0.13 | 0.00 | 0.00 | 0.35 | 0.65 | <0.001 |

**Table S3. Proportion of genotypes in males and females for SNPs with alleles that segregate with sex according to the ‘null inclusive’ sex-linked model in populations of *Niveoscincus* *ocellatus*.** Score of ‘2’ indicates heterozygous for the reference and SNP allele, ‘1’ homozygous for the SNP allele**^+^**, ‘0’ homozygous for the reference allele**^#^**,’-‘ homozygous for a null genotype. All loci denote XY heterogamety. Male genotypes of loci marked with * are ‘0’ in one population and ‘1’ in the other.

| Common  Loci | REF > SNP | Males | | | | Females | | | | p value |
| --- | --- | --- | --- | --- | --- | --- | --- | --- | --- | --- |
|  |  | Prop. ‘2’ | Prop. '1' | Prop. '0' | Prop. '-' | Prop. '2' | Prop. '1' | Prop. ‘0’ | Prop. ‘-’ |  |
| NO04145^*^ | A>T | 0.00 | 0.36 | 0.43 | 0.21 | 0.00 | 0.00 | 0.00 | 1.00 | <0.001 |
| NO04127 ^*^ | G>A | 0.00 | 0.49 | 0.51 | 0.00 | 0.00 | 0.00 | 0.00 | 1.00 | <0.001 |
| NO04162 | G>T | 0.00 | 0.96 | 0.04 | 0.00 | 0.00 | 0.00 | 0.00 | 1.00 | <0.001 |
| NO07509 ^*^ | G>T | 0.00 | 0.45 | 0.49 | 0.06 | 0.00 | 0.00 | 0.00 | 1.00 | <0.001 |
| NO07552 ^*^ | G>C | 0.00 | 0.40 | 0.45 | 0.15 | 0.00 | 0.00 | 0.00 | 1.00 | <0.001 |
| NO11008 ^*^ | C>G | 0.00 | 0.45 | 0.45 | 0.11 | 0.00 | 0.00 | 0.00 | 1.00 | <0.001 |
| NO18978 | C>G | 0.00 | 0.87 | 0.00 | 0.13 | 0.00 | 0.00 | 0.02 | 0.98 | <0.001 |
| NO24879 | G>A | 0.04 | 0.96 | 0.00 | 0.00 | 0.00 | 0.02 | 0.05 | 0.93 | <0.001 |
| NO25173 | A>C | 0.02 | 0.98 | 0.00 | 0.00 | 0.00 | 0.07 | 0.15 | 0.78 | <0.001 |
| NO25258 | G>C | 0.04 | 0.96 | 0.00 | 0.00 | 0.00 | 0.00 | 0.12 | 0.88 | <0.001 |
| NO26563 | T>C | 0.02 | 0.94 | 0.04 | 0.00 | 0.00 | 0.00 | 0.00 | 1.00 | <0.001 |
| NO26593 | C>A | 0.00 | 0.77 | 0.00 | 0.23 | 0.00 | 0.00 | 0.05 | 0.95 | <0.001 |
| NO27294 | T>G | 0.02 | 0.98 | 0.00 | 0.00 | 0.00 | 0.00 | 0.12 | 0.88 | <0.001 |
| NO27500 | T>C | 0.00 | 1.00 | 0.00 | 0.00 | 0.00 | 0.02 | 0.02 | 0.95 | <0.001 |
| NO27612 | A>G | 0.00 | 1.00 | 0.00 | 0.00 | 0.00 | 0.02 | 0.07 | 0.90 | <0.001 |
| NO04199 | T>C | 0.00 | 0.89 | 0.04 | 0.06 | 0.00 | 0.00 | 0.00 | 1.00 | <0.001 |
| NO04080 | G>C | 0.00 | 0.04 | 0.96 | 0.00 | 0.00 | 0.00 | 0.00 | 1.00 | <0.001 |
| NO04084 ^*^ | A>G | 0.00 | 0.49 | 0.43 | 0.09 | 0.00 | 0.00 | 0.00 | 1.00 | <0.001 |
| NO04117 | C>T | 0.00 | 0.06 | 0.83 | 0.11 | 0.00 | 0.00 | 0.00 | 1.00 | <0.001 |
| NO04124 ^*^ | G>A | 0.00 | 0.43 | 0.45 | 0.13 | 0.00 | 0.00 | 0.00 | 1.00 | <0.001 |
| NO07495 | G>A | 0.00 | 0.04 | 0.85 | 0.11 | 0.00 | 0.00 | 0.02 | 0.98 | 0.003 |
| NO04100 | C>T | 0.00 | 0.04 | 0.94 | 0.02 | 0.00 | 0.00 | 0.00 | 1.00 | <0.001 |
| NO08908 | C>T | 0.06 | 0.02 | 0.77 | 0.15 | 0.00 | 0.10 | 0.00 | 0.90 | <0.001 |
| NO09550 | C>T | 0.00 | 0.04 | 0.96 | 0.00 | 0.00 | 0.00 | 0.02 | 0.98 | <0.001 |
| NO12829 | T>G | 0.00 | 0.00 | 1.00 | 0.00 | 0.00 | 0.05 | 0.07 | 0.88 | <0.001 |
| NO13113 | G>A | 0.11 | 0.00 | 0.89 | 0.00 | 0.00 | 0.12 | 0.00 | 0.88 | <0.001 |
| NO13978 | G>A | 0.00 | 0.02 | 0.96 | 0.02 | 0.00 | 0.00 | 0.00 | 1.00 | <0.001 |
| NO17128 | T>A | 0.02 | 0.00 | 0.98 | 0.00 | 0.00 | 0.07 | 0.05 | 0.88 | <0.001 |
| NO21421 | C>A | 0.02 | 0.06 | 0.91 | 0.00 | 0.00 | 0.00 | 0.00 | 1.00 | <0.001 |
| NO22921 | C>A | 0.00 | 0.00 | 0.98 | 0.02 | 0.00 | 0.02 | 0.05 | 0.93 | <0.001 |
| NO25881 | A>G | 0.00 | 0.02 | 0.91 | 0.06 | 0.00 | 0.02 | 0.00 | 0.98 | <0.001 |
| NO25172 | C>A | 0.02 | 0.00 | 0.98 | 0.00 | 0.00 | 0.07 | 0.07 | 0.85 | <0.001 |
| NO27502 | G>A | 0.00 | 0.00 | 1.00 | 0.00 | 0.00 | 0.05 | 0.02 | 0.93 | <0.001 |
| Highland  Loci | REF > SNP | Males | | | | Females | | | | p value |
|  |  | Prop. ‘2’ | Prop. '1' | Prop. '0' | Prop. '-' | Prop. '2' | Prop. '1' | Prop. ‘0’ | Prop. ‘-’ |  |
| NO10275 | C>A | 0.00 | 0.70 | 0.00 | 0.30 | 0.00 | 0.05 | 0.00 | 0.95 | 0.026 |
| NO26341 | G>A | 0.00 | 1.00 | 0.00 | 0.00 | 0.00 | 0.00 | 0.00 | 1.00 | <0.001 |
| NO27242 | G>C | 0.13 | 0.70 | 0.04 | 0.13 | 0.00 | 0.00 | 0.29 | 0.71 | <0.001 |
| NO19405 | C>A | 0.00 | 0.00 | 0.91 | 0.09 | 0.00 | 0.00 | 0.00 | 1.00 | <0.001 |
| NO26343 | T>A | 0.04 | 0.00 | 0.96 | 0.00 | 0.00 | 0.24 | 0.00 | 0.76 | <0.001 |
| NO26549 | C>G | 0.00 | 0.00 | 1.00 | 0.00 | 0.00 | 0.00 | 0.05 | 0.95 | <0.001 |
| Lowland  Loci | REF > SNP | Males | | | | Females | | | | p value |
|  |  | Prop. ‘2’ | Prop. '1' | Prop. '0' | Prop. '-' | Prop. '2' | Prop. '1' | Prop. ‘0’ | Prop. ‘-’ |  |
| NO07823 | C>T | 0.00 | 0.67 | 0.04 | 0.29 | 0.00 | 0.00 | 0.00 | 1.00 | 0.003 |
| NO15685 | T>C | 0.00 | 0.92 | 0.00 | 0.08 | 0.00 | 0.00 | 0.00 | 1.00 | <0.001 |
| NO16129 | G>A | 0.00 | 0.92 | 0.00 | 0.08 | 0.00 | 0.00 | 0.05 | 0.95 | <0.001 |
| NO25994 | A>T | 0.00 | 0.67 | 0.04 | 0.29 | 0.00 | 0.00 | 0.15 | 0.85 | 0.012 |
| NO26367 | A>G | 0.00 | 1.00 | 0.00 | 0.00 | 0.00 | 0.00 | 0.05 | 0.95 | <0.001 |

**^#+^** Given the prevalence of null alleles in females it is accepted that individuals with a ‘0’ or ‘1’ genotype are in fact heterozygous for a null allele and the reference or SNP allele respectively.
